# FcgRIII Deficiency and FcgRIIb Defeciency Promote Renal Injury in Diabetic Mice

**DOI:** 10.1155/2019/3514574

**Published:** 2019-08-22

**Authors:** Rui Zhang, Tingli Wang, Qinhua Yin, Junlin Zhang, Li Li, Ruikun Guo, Qianqian Han, Hanyu Li, Yiting Wang, Jiali Wang, Pramesh Gurung, Yanrong Lu, Jingqiu Cheng, Lin Bai, Jie Zhang, Fang Liu

**Affiliations:** ^1^Division of Nephrology, West China Hospital of Sichuan University, Chengdu 610041, Sichuan, China; ^2^Key Laboratory of Transplant Engineering and Immunology, Ministry of Health, Regenerative Medicine Research Center, West China Hospital of Sichuan University, Chengdu 610041, Sichuan, China

## Abstract

The immune system is involved in the development of diabetes complications and IgG Fc gamma receptors (FcgRs) are key immune receptors responsible for the effective control of both humoral and innate immunity. We investigated the effects of members of the FcgR superfamily into both the streptozotocin plus high fat-induced type 2 diabetes and high fat diet (HFD) models. FcgRIII^−/−^ diabetic mice and FcgRIIb^−/−^ diabetic mice had elevated levels of serum creatinine compared with wildtype (WT) diabetic mice. Renal histology of diabetic FcgRIII knockout and FcgRIIb knockout mice showed mesangial expansion and GBM thickening; the mechanistic study indicated a higher expression of TGF-*β*1, TNF-*α*, and p-NF*κ*B-p65 compared with wild type mouse. The HFD mouse with FcgRIII knockout or FcgRIIb knockout had increased biochemical and renal injury factors, but oxLDL deposition was higher than in FcgRIII^−/−^ diabetic mice and FcgRIIb^−/−^ diabetic mice.* In vitro* we further examined the mechanism by which the Fc gamma receptor promoted renal injury and transfected glomerular mesangial cells (GMCs) with FcgRI siRNA attenuated the level of TGF-*β*1, TNF-*α* expression. In summary, FcgRI knockdown downregulated kidney inflammation and fibrosis and FcgRIIb knockout accelerated inflammation, fibrosis, and the anomalous deposition of oxLDL whereas FcgRIII deficiency failed to protect kidney from diabetic renal injury. These observations suggested that FcgRs might represent a novel target for the therapeutic intervention of diabetic nephropathy.

## 1. Introduction

The past few decades have witnessed a marked increase in the prevalence of diabetic nephropathy (DN), which develops in approximately 30% of patients with type 1 diabetes mellitus (T1DM) and 40% of patients with type 2 diabetes mellitus (T2DM)[1, 2]. DN is now the leading cause of end-stage renal disease (ESRD) in developed countries and is more common than chronic kidney disease (CKD) associated glomerulonephritis in China [3, 4]. The management of common risk factors, such as oxidative stress, inflammatory infiltration, hyperglycemia, hyperlipidemia and hypertension has reduced the rates of incident diabetic cardiovascular complications in recent years, although there seems to be a rare effect on the prevalence of diabetic kidney diseases [5]. Given the huge healthcare burden related to DN, it is important to identify specific risk factors and the underlying mechanisms involved in this common diabetic microvascular complication.

High glucose and hypercholesterolemia are recognized as the two main characteristics of diabetes mellitus as well as important risk factors for the occurrence and development of DN. Oxidative LDL (oxLDL) is immunogenic and induces autoantibody production, which increases the formation of LDL-containing immune complexes [6]. Overproduction of oxLDL because of hypercholesterolemia, increased the ectopic deposition of oxidative LDL immune complex (ox-LDL-IC) in glomerular mesangial areas. The deposition of immunoglobulin G (IgG) triggers proinflammatory and profibrosis events and contributes to the progression of microalbuminuria, macroalbuminuria and glomerulosclerosis in DN, termed renal lipotoxicity [7]. Furthermore, previous studies reported that ox-LDL-IC led to the overexpression of collagen IV in mesangial cells through Fc gamma receptor III (FcgRIII), which implied the involvement of FcgRIII in ox-LDL-ICs induced renal fibrosis [8].

Accumulating evidence suggests that the immune system is involved in the development of diabetes complications. IgG immune complexes are recognized by infiltrating and resident cells through specific receptors of the Fc region [9, 10]. The Fc gamma receptors (FcgRs) are immune receptors that have an important role in the clearance of immune complexes and the presentation of complexed antigens. FcgRs are a family of proteins expressed by a variety of immune cells [11], implementing their biological functions by binding to the Fc region of IgG for the effective control of inflammation and responses to infection. In mice, four different classes of FcgRs have been characterized: FcgRI/CD64, FcgRIIb/ CD32, FcgRIII/ CD16, and FcgRIV [12–14], which differ by their distinct affinity, cellular distributions, and biological functions.

This study investigated the involvement of FcgR members (FcgRI, FcgRIIb, and FcgRIII) in high glucose and ox-LDL-IC-induced fibrosis and inflammatory injury in diabetic mice.

## 2. Research Design and Methods

### 2.1. In Vivo Studies

#### 2.1.1. Diabetic Animal Model of FcgRIII Knockout and FcgRIIb Knockout Mice

Animal experiments were performed with the approval of the Sichuan University Animal Ethics Committee. FcgRIII (CD16) knockout mice (FcgRIII^−/−^, C57BL/6J background) and FcgRIIb (CD32) knockout mice (FcgRIIb^−/−^, C57BL/6J background) were purchased from the Jackson Laboratory and C57BL/6J wild-type mice ( WT, male, aged 8 weeks, 20–25 g ) were purchased from the Laboratory Animal Centre of Sichuan University. All mice were housed simultaneously in stainless steel wire cages with controlled temperature (22 ± 2°C) and relative humidity of 40%–60% and maintained on a reverse 12-h dark (7:00 a.m. to 7:00 p.m.) and light (7:00 p.m. to 7:00 a.m.) cycle in the animal facilities of the Laboratory Animal Centre of Sichuan University.

Diabetic models were induced in littermate FcgRIII^−/−^, FcgRIIb^−/−^ and WT mice (male, aged 8 weeks, 20–30 g). Diabetic mice models were fed a high-fat diet (HFD, containing regular diet plus 27.3% lard, 54.6% sucrose, 16.4% cholesterol, and 1.6% sodium cholate from Beijing KeaoXieli Feed Co. Ltd., Beijing, China) and then injected with multiple doses of low-dose streptozotocin (STZ) (four doses of 55 mg/kg; Sigma, St. Louis, MO, USA) in citrate buffer (pH 4.5) after overnight fasting. The control mice were injected with citrate buffer (1 ml/kg). The fasting blood glucose (FBG) of the mice was determined using a glucometer (Accu-Chek, Roche). Mice with an FBG >16.7 mmol/L for consecutive 3 days were considered diabetic group and were used for this study. High-fat diet mice and control mice were kept in the same period diabetic group. The mice were maintained on their respective diets until the end of the study.

The mice were then divided into the following groups: C57BL/6J WT with normal diet (n=6), C57BL/6J WT with HFD (n=6), C57BL/6J WT with diabetes (n=6); FcgRIII^−/−^ with normal diet (n=6), FcgRIII^−/−^ with HFD (n=6), FcgRIII^−/−^ with diabetes (n=6); FcgRIIb^−/−^ with normal diet (n=6), FcgRIIb^−/−^ with HFD (n=6), and FcgRIIb^−/−^ with diabetes (n=6). Groups of six mice were euthanized on week 10 after STZ or citrate buffer injection. Samples of the renal cortex were collected for histology, immunohistochemistry, western blot, and real-time PCR analyses.

#### 2.1.2. Biochemical Measurements

Prior to sacrifice, blood glucose was measured after a 6-h daytime fast using a glucose analyzer. Blood samples were obtained by cardiac puncture. Clinical biochemical analysis was performed using an autoanalyzer (Cobas Integra 400 Plus, Roche, Basel, Switzerland) using commercial kits and the following parameters were measured: fasting blood glucose (FBG), total cholesterol (TC), low-density lipoprotein (LDL), blood urea nitrogen (BUN), and serum creatinine (CRE).

#### 2.1.3. Isolation of Glomeruli

The method of glomeruli isolation was based on the protocol described by Minoru Takemoto in 2002[15]. Mice were anesthetized by an intraperitoneal injection of chloral hydrate and perfused with Dynabeads diluted in Hanks' buffered salt solution (HBSS) through the heart. The kidneys were removed after a few time, cut into pieces and digested in collagenase A for 30 min. The collagenase-digested tissue was gently pressed respectively through a 100-*μ*m, 70-*μ*m, 40-*μ*m cell strainer using a flattened pestle. The filtered glomeruli were collected in 40-*μ*m cell strainer washed with HBSS. Isolated glomeruli containing Dynabeads were gathered by a magnetic particle concentrator. We used acridine orange fluorescent staining to mark the isolated glomeruli ([Supplementary-material supplementary-material-1]).

#### 2.1.4. Renal Histologic Analysis

Renal tissues were fixed in 10% neutral buffered formaldehyde or 4% paraformaldehyde, embedded in paraffin and then 4 *μ*m sections were cut and stained with standard periodic acid–Schiff, Masson's trichrome and histological techniques for light microscopic examination[16, 17]. For immunohistochemical staining, primary antibodies against Anti-TGFBI (1:400, ab170874, Abcam), Anti-TNF alpha (1:400, ab1793, Abcam), Anti-NF-kB p65 (phospho S536) (1:600, ab86299, Abcam), and Anti-oxLDL (1:600, ab14519, Abcam) was used. The samples were then stained with ChemMate^TM^ Envision+HRP (Envision^TM^ Detection Kit, Code No: GK500705, Gene Tech, Shanghai) and incubated for 45 min at 37°C. Sections were then stained with diaminobenzidine (DAB) and counterstained with hematoxylin. In six mice per group, more than 20 cortical glomeruli were assessed for each mouse. The positive areas were quantified by Image-Pro Plus version 6.0 software according to methods previously reported [18, 19].

#### 2.1.5. Electron Microscopy

Tissue samples of the kidney were examined using an transmission electron microscope (TEM), the measurement of the width of glomerular basement membrane (GBM) was determined based on the methods described [20, 21].

#### 2.1.6. Immunofluorescence

For immunofluorescence (IF) staining, 4 mm sections of freshly frozen kidney tissue were fixed in 4% paraformaldehyde, washed in PBS for 30 min and incubated with the following primary monoclonal antibodies: Anti-TGFBI (1:200, ab170874, Abcam), Anti-TNF alpha (1:200, ab1793, Abcam), Anti-NF-kB p65 (phospho S536) (1:300, ab86299, Abcam), and Anti-oxLDL (1:300, ab14519, Abcam). The sections were washed with PBS, incubated with diluted fluorescence-conjugated secondary antibodies including donkey anti-rabbit IgG/FITC (Merck Millipore, Billerica, MA, USA) at 37°C in the dark for 1 h, and then stained with DAPI (Calbiochem). A micrograph of stained sections was acquired using a confocal microscope (Fluoview1000, Olympus, Tokyo, Japan) with FV10-ASW software (version 1.7, Olympus).

### 2.2. In Vitro Studies

#### 2.2.1. Cell Culture of Glomerular Mesangial Cells (GMCs)

Well-characterized normal glomerular mesangial cells (GMCs) (SV40-MES-13, ATCC) were used in this study and were cultured in DMEM at 37°C in a 5% CO_2_ atmosphere. Cells were harvested with 0.25% trypsin (Gibco) at approximately 80% confluence, and the cells were used for experiments within six passages. GMCs were transferred to serum-free medium 12 h prior to treatment, which was then replaced by DMEM with normal glucose (NG, 5 mmol/L), high glucose (HG, 25 mmol/L), high glucose plus oxLDL (HG, 25 mmol/L and oxLDL 1 mg/mL), respectively for 24 h. Cells were then harvested with 0.25% trypsin (Gibco, Life Technologies, Carlsbad, CA, USA) for further analysis by real-time PCR and western blot.

#### 2.2.2. Preparation of ox-LDL-IC

Insoluble ox-LDL-IC was obtained by polyethylene glycol precipitation as previously reported [19, 22, 23]. In brief, C57BL/6J mice were immunized with human oxidized low-density lipoprotein (ox-LDL, 1 mg/mL, Sigma, USA) 4 times with 30 *μ*g per injection. Then, the IgG was purified from the serum by affinity chromatography and mixed with varying amounts (50–500 *μ*g) of ox-LDL at 4°C overnight. Polyethylene glycol (PEG) in borate-buffered saline, pH 8.4, was added drop by drop until the final PEG concentration was 4%. After overnight incubation at 4°C, 10 *μ*l cesium chloride was added to each sample and centrifuged at 1509.3xg for 20 min to identify the immune complex by density gradient centrifugation. According to a precipitin curve, human ox-LDL (1 mg/mL) was mixed with specific mouse IgG at a ratio of 1.0:0.5.

#### 2.2.3. Short Hairpin (si)RNA Transfection

GMCs were plated in 6-well plates and flasks and maintained in DMEM with 5% fetal bovine serum. Lentivirus vectors carrying siRNA targeting FcgRI, FcgRIIb and/or FcgRIII RNAs were purchased from Saierbio Technology Incorporation (Tianjin, China) and were respectively transferred into GMCs. The transferred cells were screened with validated FcgRI-siRNA, FcgRIIb-siRNA and/or FcgRIII-siRNA puromycin to identify those with stable silencing gene expression. Silencer negative control siRNA and silencer FcgRI-siRNA, FcgRIIb-siRNA and/or FcgRIII-siRNA were purchased from System Bioscience (Shanghai, China). Transfection with the respective FcgR siRNAs diluted in Opti-MEM I (100 nmol/L) was performed using Lipofectamine 2000 Reagent (Invitrogen, USA) according to the manufacturer's protocol. The culture medium was changed 24 h after transfection. After transfection, the cells were incubated with normal glucose and high glucose plus oxLDL for 24 h before cell supernatants and cells were collected for real-time PCR and western blot analysis.

### 2.3. RNA Isolation and Real-Time PCR Analysis

Total RNA from glomeruli or cultured cells was extracted using TRIzol reagent (Thermo Scientific Inc., MA, USA) and its concentration was measured on a microspectrophotometer (Thermo Scientific Inc., MA, USA). The RNA quality was tested by agar gel electrophoresis followed by cDNA synthesis. cDNA was amplified from RNA using a commercial kit (Bio-Rad, Hercules, CA, USA). Primer sequences are shown in [Supplementary-material supplementary-material-1]. Real-time PCR was performed with the CFX96TM Real-Time System (Bio-Rad, Hercules, USA) using SYBR Premix Ex Taq™ (Tli RNaseH Plus Takara) as previously described [16]. The housekeeping gene* GAPDH *or* β-actin *was used as an internal standard. PCR reactions were carried out in a volume of 20 ml on a CFX96 RealTime PCR System (Bio-Rad) with SYBR Green kit (Tli RNaseH Plus, Takara), followed by melting curve analysis to distinguish the specific and non-specific PCR products. The relative expression of each gene was calculated using the 2^−ΔΔCt^ method.

### 2.4. Western Blot Analysis

Total protein extraction was performed as previously described [18]. The protein concentration was determined by the BCA method. Proteins were separated by SDS-PAGE and then transferred to PVDF membranes (0.45 mm, Millipore). The PVDF membranes were washed with TBST, blocked for 1 h with 5% skim milk powder dissolved in TBST and incubated with primary antibodies against TGF-*β* (ab170874, Abcam), TNF-*α* (ab1793, Abcam), p-NFKB (ab86299, Abcam), and oxLDL (ab14519, Abcam) at 4°C overnight with dilutions recommended by the manufacturer. GAPDH was used as an internal reference. The PVDF membranes were washed with TBST and incubated with horseradish peroxidase (HRP) conjugated secondary antibodies at 37°C for 1 h. Protein bands were detected using chemiluminescence (ECL) reagent (Pierce, Thermo Scientific). The quantitative analysis of protein band density was performed on Quantity One (Bio-Rad).

### 2.5. Statistical Analysis

Statistics were presented as the mean ± SEM. SPSS software (version 21, IBM Corp., NY, USA) was performed for statistical analysis. Comparisons among groups were tested by one-way analysis of variance (ANOVA) followed by with the Bonferroni adjustment/Tukey test or the Dunnett T3 test. P values < 0.05 was considered statistically significant

## 3. Results

### 3.1. Biochemical Data of Diabetic Mice and Nondiabetic Controls

We measured and compared the biochemical data of FcgRIII^−/−^ and FcgRIIb^−/−^ diabetic mice and WT mice were used as control group (Table 1). The data demonstrated that the mean levels of blood glucose, LDL, Chol and body weight were higher in FcgRIII^−/−^ or FcgRIIb^−/−^ diabetic mice compared with the WT diabetic mice, which implies the beneficial effect of FcgRIII and FcgRIIb on carbohydrate and cholesterol metabolism. Serum creatinine and urea in FcgRIII^−/−^ and FcgRIIb^−/−^ diabetic mice were higher than in WT diabetic mice, which might suggest the renoprotective role of FcgRIII or FcgRIIb. However, the clinical and biochemical characteristics of FcgRIII^−/−^ diabetic mice showed no significant difference with FcgRIIb^−/−^ diabetic mice.

### 3.2. Glomerular or Cortical Histopathological Alteration of Diabetic Mice

**Figure 1:**
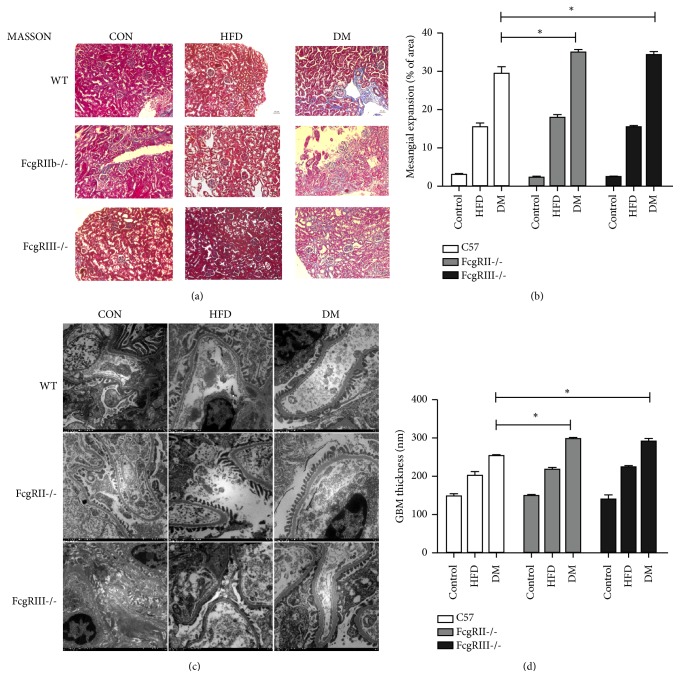
*Glomerular or cortical histopathology and ultrastructural alteration.* (a) Masson's trichrome staining of kidney tissues from different mice groups (scale bar, 50 um). (b) Semiquantitative analyses of the glomerular mesangial expansion. (c) Electron micrographs in glomerular ultrastructural alteration. (d) Glomerular basement membrane thickness. *∗*p < 0.05.

Pathomorphology of renal tissue was performed to evaluate morphological alterations in the different groups. Diabetic mice had morphological changes including GBM thickening, mesangial expansion and glomerulosclerosis. Masson staining showed that there was more apparent extracellular matrix deposit in the glomeruli of diabetic FcgRIII^−/−^ and FcgRIIb^−/−^ diabetic mice (Figures 1(a) and 1(b)).

### 3.3. Glomerular Basement Membrane Thickness in Diabetic Mice

To observe glomerular ultrastructure, transmission electron microscope was applied to observe the ultrastructure. Obviously thickening of GBM and fusion of podocyte foot process was detected in diabetic mice. Furthermore, the width of GBM was measured in FcgRIII^−/−^ and FcgRIIb^−/−^ diabetic mice (Figures 1(c) and 1(d)). The results showed that average width of GBM in FcgRIII^−/−^ diabetic mice and FcgRIIb^−/−^ diabetic mice were 300.21±11.78 nm and 294.55±4.87 nm which were greater than that in diabetic WT mice (251.76±6.04 nm). The impairment of the glomerular filtration barrier is consistent with the even worse albuminuria in the diabetic nephropathy.

### 3.4. Different Expressions of FcgRs (FcgRI, FcgRIIb and FcgRIII) in the Isolated Renal Glomeruli of Diabetic Mice

**Figure 2:**
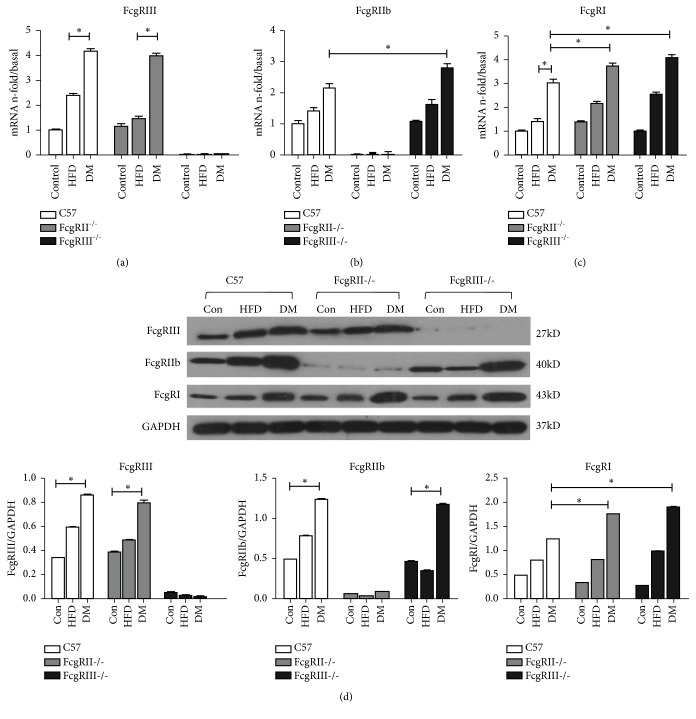
*Expressions of Fcgamma receptors in different mouse groups.* Expressions of activating FcgRs (types I and III) and inhibitory FcgRIIb in isolated renal glomeruli were detected by real-time PCR (a, b, c) and Western blot (d). *∗p* < 0.05.

To determine the different roles of the FcgR subtypes in the regulation of inflammatory responses, we examined the expressions of FcgRI, FcgRIIb, and FcgRIII in isolated renal glomeruli. Real-time PCR analysis (Figures 2(a), 2(b), and 2(c)) demonstrated increased mRNA levels of FcgRI and FcgRIII (activating receptors) and FcgRIIb (inhibitory receptor) in WT diabetic mice. In the glomeruli of FcgRIII^−/−^ diabetic mice, the expressions of FcgRIIb and FcgRI were increased significantly compared with WT diabetic mice, and the expressions of FcgRI were higher in the glomeruli of FcgRIIb^−/−^ diabetic mice compared with WT diabetic mice. Moreover, western blot (Figure 2(d)) for protein levels were not entirely consistent with transcriptional level, that FcgRIII-/- and FcgRIIb-/- diabetic mice exhibited significantly increased FcgRI. The relation between mRNA and protein is not strictly linear. The different expressions of the FcgR subtypes in the glomeruli of diabetic mice suggest they have different roles in the pathogenesis of DN.

### 3.5. Role of FcgRIII or FcgRIIb in the Inflammatory and Fibrosis of Glomeruli in Diabetic Mice

**Figure 3:**
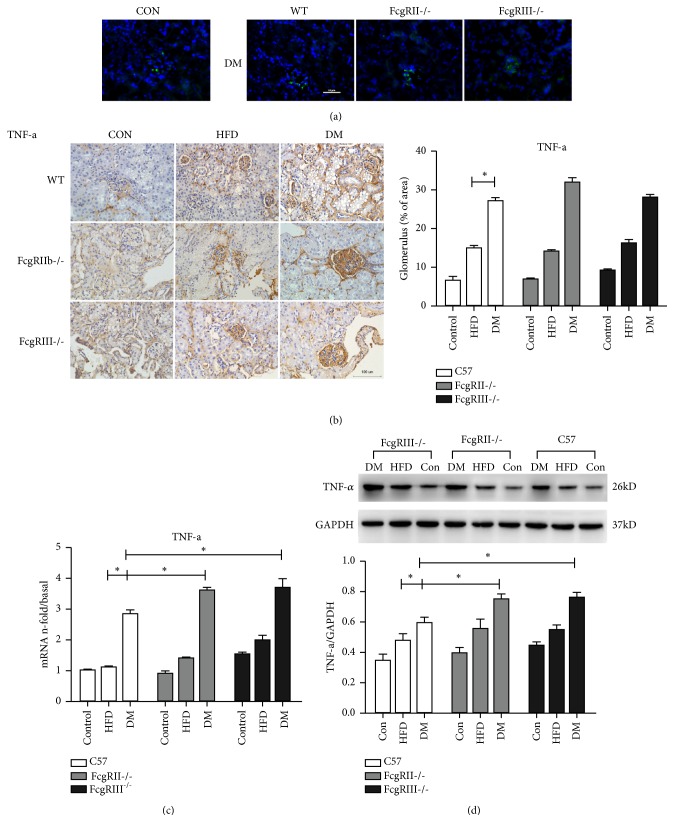
Effect of FcgRIII or FcgRIIb deficiency on renal inflammation in diabetic mice. (a) Immunofluorescence staining for TNF-*α* (scale ×200). (b) Immunohistochemistry staining of TNF-*α* and quantification (scale bar = 100 *μ*m). (c) The mRNA expression of TNF-*α* was measured by real-time PCR, *∗p* < 0.05. (d) Western blot of TNF-*α* expression. *∗p* < 0.05.

To investigate the roles of FcgRIII and FcgRIIb in inflammatory injury in glomeruli, we detected the expressions of TNF-*α* and NF-*κ*B-p65 by immunohistochemistry, immunofluorescence and western blot analysis. Immunofluorescence (Figure 3(a)) and immunohistochemistry (Figure 3(b)) demonstrated a higher expression of TNF-*α* in the diabetic mice compared with the control mice (both WT diabetic mice and FcgRIII^−/−^ or FcgRIIb^−/−^ diabetic mice; Figures 3(a) and 3(b)). Moreover, western blot and real-time PCR revealed the expression of TNF-*α* in glomeruli of FcgRIII^−/−^ and FcgRIIb^−/−^ diabetic mice was higher than in the HFD and WT control groups (Figures 3(c) and 3(d)); however, TNF-*α* expression was comparable in the FcgRIII^−/−^ and FcgRIIb^−/−^ groups.

**Figure 4:**
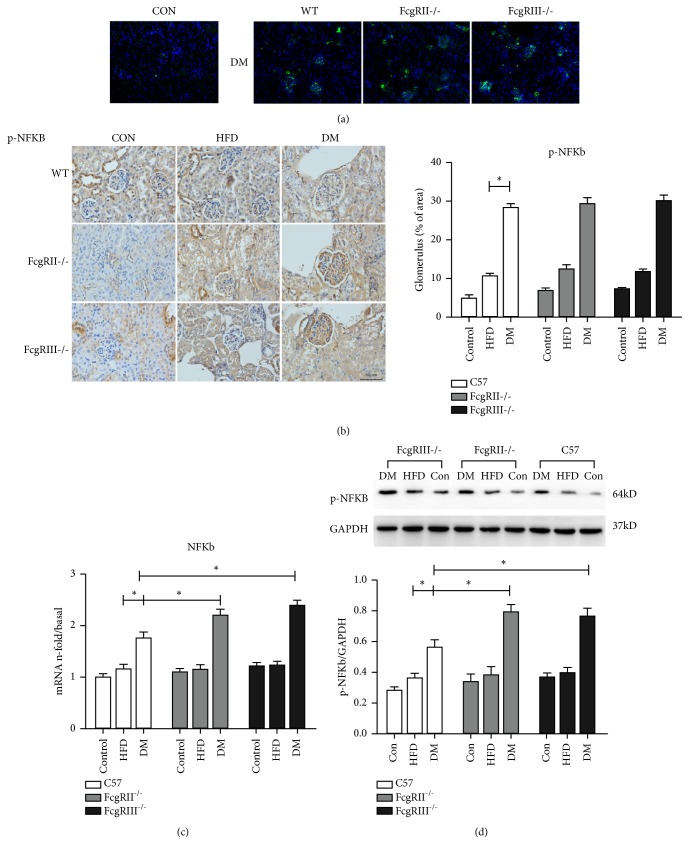
*Effect of FcgRIII or FcgRIIb deficiency on NKκB-p65 expression in diabetic mice.* (a) Immunofluorescence staining of p-NF*κ*B-p65 (scale ×200). (b) Immunohistochemistry staining of p-NF*κ*B-p65 and quantification (scale bar = 100 *μ*m). (c) mRNA expression of NF*κ*B-p65 was measured by real-time PCR, *∗p *< 0.05. (d) Western blot of p-NF*κ*B-p65 expression, *∗p *< 0.05.

Moreover, immunohistochemistry and immunofluorescence showed the stronger nuclear activation of p-NF-*κ*B in the glomeruli of diabetic FcgRIII^-/-,^ FcgRIIb^−/−^ and WT mice, compared with normal control mice and HFD mice, (Figure 4). Western blot and real-time PCR also demonstrated the higher expression of NF-*κ*B in the glomeruli of diabetic mice compared with normal control mice and HFD mice; however, there were no significant differences between FcgRIII^−/−^ and FcgRIIb^−/−^ mice.

**Figure 5:**
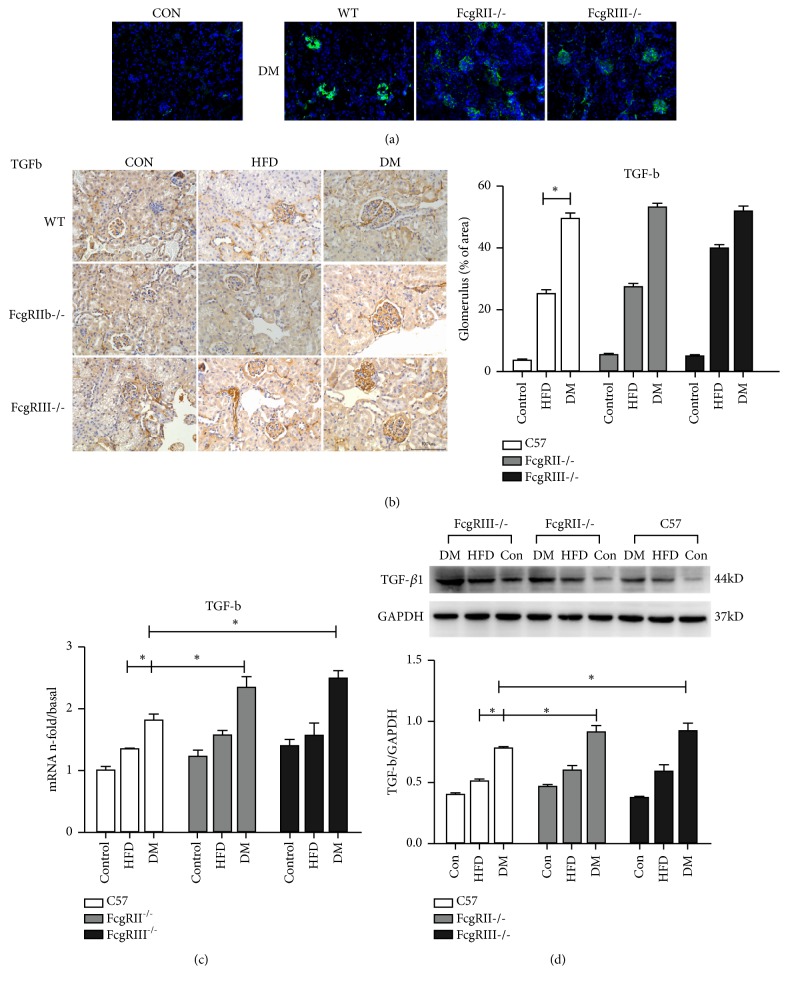
*Effects of FcgRIII or FcgRIIb deficiency on renal fibrosis in diabetic mice.* (a) Immunofluorescence staining of TGF-*β* (scale ×200). (b) Immunohistochemistry staining of TGF-*β* and quantification (scale bar = 100 *μ*m). (c) mRNA expression of TGF-*β* was measured by real-time PCR, *∗p* < 0.05. (d) Western blot of TGF-*β* expression, *∗p* < 0.05.

We investigated the fibrosis in glomeruli by detecting the expression of TGF-*β*1, which promotes extracellular matrix accumulation and induces renal fibrosis. As shown in Figure 5, the results of the fibrosis in glomeruli of diabetic mice were in accordance with the trend of inflammatory injury. Immunohistochemistry and immunofluorescence demonstrated that TGF-*β*1 was strong positive in the glomeruli of diabetic mice and HFD mice, while normal mice had a lower TGF-*β*1 expression. Western blot and real-time PCR for TGF-*β*1 was increased in the glomeruli of diabetic mice, particularly FcgRIII^−/−^ and FcgRIIb^−/−^ diabetic mice. The expression of TGF-*β*1 in the glomeruli of FcgRIII^−/−^ and FcgRIIb^−/−^ diabetic mice were higher than in WT diabetic mice. Furthermore, the expression of TGF-*β*1 in the glomeruli was no significantly difference between FcgRIII^−/−^ and FcgRIIb^−/−^ mice (Figures 5(c) and 5(d)).

### 3.6. Expression of oxLDL in the Glomeruli of Diabetic Mice

**Figure 6:**
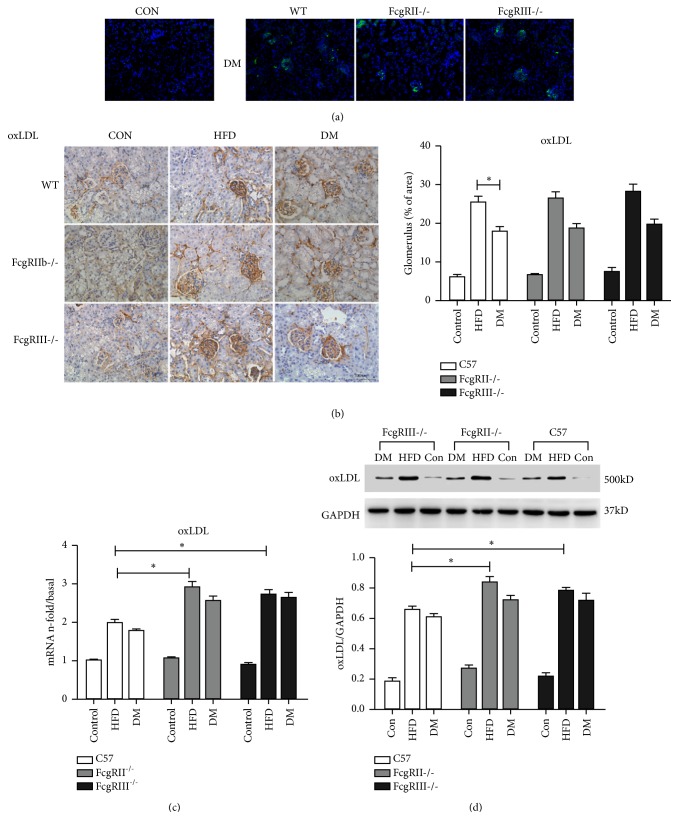
*Effect of FcgRIII or FcgRIIb deficiency on the expression of oxLDL in diabetic mice.* (a) Immunofluorescence staining of oxLDL (scale ×200). (b) Immunohistochemistry staining of oxLDL and quantification (scale bar = 100*μ*m). (c) mRNA expression of oxLDL was measured by real-time PCR, *∗p *< 0.05. (d) Western blot of oxLDL expression. *∗p *< 0.05.

We measured the expression of oxLDL in the glomeruli by immunohistochemistry, immunofluorescence, western blot and real-time PCR. As shown in Figures 6(a) and 6(b) by immunohistochemistry and immunofluorescence, oxLDL expression was higher in the glomeruli of diabetic mice and especially HFD mice when compared with WT normal mice, which had very weak oxLDL staining. Furthermore, the mRNA and protein expressions of oxLDL in the glomeruli were higher in FcgRIII^−/−^ HFD mice and FcgRIIb^−/−^ HFD mice compared with WT HFD mice (Figures 6(c) and 6(d)).

### 3.7. The Relationship between FcgRI, FcgRIIb, and FcgRIII in Mesangial Cells under the Conditions of High Glucose and oxLDL-IC

**Figure 7:**
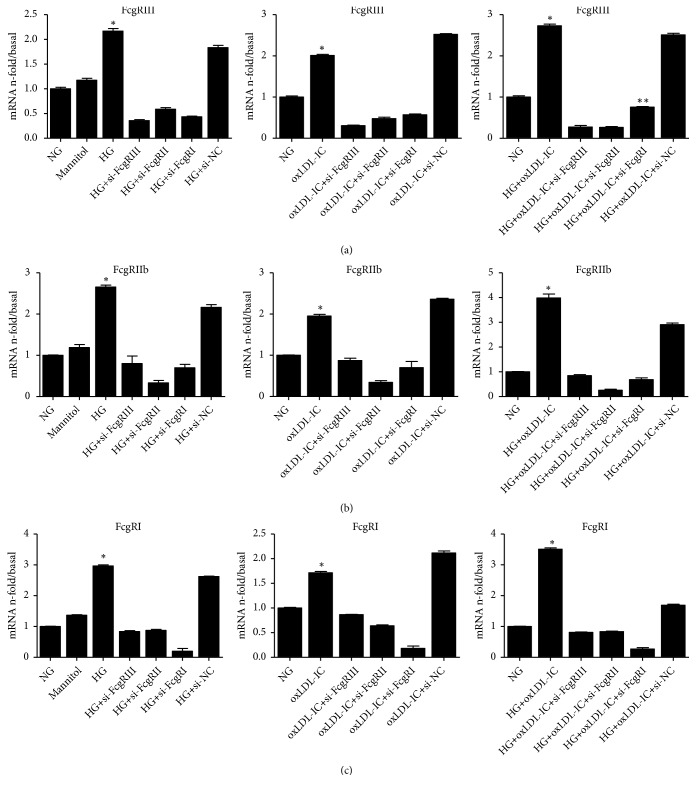
*Relationship among FcgRI, FcgRIIb and FcgRIII in GMCs under the conditions of high glucose and oxLDL-IC.* GMCs were cultured with normal glucose (5 mmol/l), high glucose (25 mmol/l), oxLDL-IC, or high glucose combined with oxLDL- IC. The mRNA expressions of FcgRIII (a), FcgRIIb (b) and FcgRI (c) were measured by real-time PCR. Data are expressed as the mean** ±** S.E. *∗p* < 0.05 versus NG; *∗∗p* < 0.05 versus HG + oxLDL- IC + siRNA-FcgRIIb.

**Figure 8:**
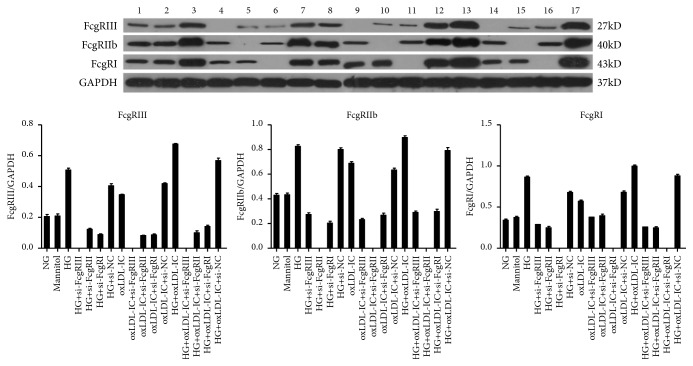
*Protein level among FcgRI, FcgRIIb and FcgRIII in GMCs under the conditions of high glucose and oxLDL-IC.* (1) normal glucose, (2) Mannitol, (3) high glucose, (4) high glucose + si-FcgRIII, (5) high glucose + si-FcgRIIb, (6) high glucose + si-FcgRI, (7) high glucose + si-NC, (8) oxLDL-IC, (9) oxLDL-IC + si-FcgRIII, (10) oxLDL-IC + si-FcgRIIb, (11) oxLDL-IC + si-FcgRI, (12) high glucose + si-NC, (13) high glucose + oxLDL-IC, (14) high glucose + oxLDL-IC + si-FcgRIII, (15) high glucose + oxLDL-IC + si-FcgRIIb, (16) high glucose + oxLDL-IC + si-FcgRI, (17) high glucose + oxLDL-IC + si-NC.

To determine which FcgR mediated injury for the most part in diabetic glomerular mesangial cells, GMCs transfected with siRNAs targeting FcgRI, FcgRIIb and FcgRIII were stimulated with high glucose or oxLDL-IC, and the expressions of FcgRI, FcgRIIb and FcgRIII were detected. The results showed that high glucose, oxLDL-IC and high glucose plus oxLDL-IC increased the expressions of FcgRI, FcgRIIb and FcgRIII compared with normal glucose (Figures 7 and 8), the group of single oxLDL-IC stimulating cell revealing the lower increasing. Meanwhile, silencing single FcgR downregulated the expression of the remaining receptors even if stimulated by high glucose or oxLDL-IC.

**Figure 9:**
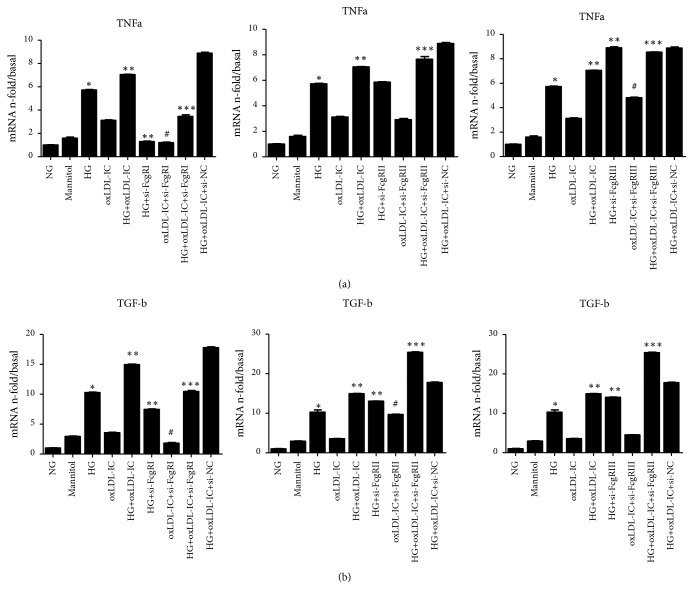
*Effects of siRNA-FcgRs on inflammation and fibrosis in GMCs.* GMCs transfected with siRNAs targeting FcgRI, FcgRIIb or FcgRIII were cultured with normal glucose (5 mmol/l), high glucose (25 mmol/l), oxLDL-IC, or high glucose combined with oxLDL-IC. The mRNA expressions of TNF-*α* (a) and TGF-*β* (b) were measured by real-time PCR. Data are expressed as the mean** ±** SEM.*∗p* < 0.05 versus NG; *∗∗p *< 0.05 versus HG; *∗∗∗p *< 0.05 versus HG + oxLDL; #*p* < 0.05 versus oxLDL.

Incubation of cells with high glucose or oxLDL-IC aggregates increased the expression of inflammation and fibrosis, the group with FcgRI siRNAs appeared declining trend of inflammation and fibrosis. As shown in Figure 9, the expressions of TNF-*α* and TGF-*β* upregulated by high glucose plus oxLDL-IC were significantly downregulated by FcgRI siRNA transfection and no obviously suppressed by FcgRII or FcgRIII siRNA transfection. These results suggest that siRNA-FcgRI significantly downregulated the inflammatory and fibrosis of GMCs under high glucose and oxLDL-IC conditions.

## 4. Discussion

Among patients with diabetes, there is strong evidence supporting a key role for the adaptive immune response in the pathogenesis of diabetic complications such as atherosclerosis and diabetic nephropathy [24, 25]. Previous studies reported that Fcg receptor deficiencies alleviated renal hypertrophy, oxidative stress, inflammation and fibrosis in hypercholesterolemic mice with diabetes, and exerted renoprotective effects on diabetic nephropathy [26–28]. However, there is no evidence to clarify the individual role of FcgR family members in the pathogenesis of diabetic nephropathy. The current study investigated the involvement of FcgRI, FcgRIIb, and FcgR III in diabetic renal injury in hyperglycemic and hypercholesterolemic FcgRIIb^−/−^ and FcgRIII^−/−^ mice. Immunohistochemistry manifested distribution in biological tissues, level of protein quantification was more accuracy by western blot, real-time PCR detected mRNA at the level of transcription. Although the expressions of FcgRI, FcgRIIb and FcgRIII were upregulated in the glomeruli of diabetic mice, FcgRI expression was higher in both FcgRIIb^−/−^ diabetic mice and FcgRIII^−/−^ diabetic mice. Moreover, both inflammatory and fibrosis in glomeruli were more severe in the FcgRIIb^−/−^ diabetic mice and FcgRIII^−/−^ diabetic mice by western blot and real-time PCR. In our study the control IHC sections of the WT appeared vacuole, the short-term mass liquids with Dynabeads might change osmotic pressure, mice was perfused with Dynabeads diluted in HBSS through the heart.

Four different subtypes of FcgRs have been identified as expressed in mouse kidney, especially the glomerular mesangial cells [13, 29, 30]. To confirm the different biological roles of FcgRs in intrinsic glomerular mesangial cells, we used Lentivirus vectors carrying siRNAs targeting the FcgRI, FcgRIIb, and FcgR III RNAs to transfect GMCs under high glucose or oxLDL-IC stimulation. The same part sequence of silence existing made an explanation that silencing single FcgR downregulated the expression of the remaining receptors. Our* in vitro* study observed that the expressions of inflammatory and fibrosis were significantly downregulated by siRNA-FcgRI, but the mRNA expression of TGF-b and TNF-a were no obviously downregulated by siRNA-FcgRIIb or siRNA-FcgRIII. This might imply that FcgRIII or FcgRIIb deficiency exacerbates renal inflammation and fibrosis, which was mainly attributable to oxidative LDL immune complex IgG binding to FcgRI in diabetic mice.

FcgR bind with varying affinity and specificity to different IgG subclass, the complexity in the FcgR family is mirrored by the presence of four different IgG subclasses in mice (IgG1, IgG2a, IgG2b and IgG3)[31]. FcgRI is a high-affinity activating receptor capable of binding to monomeric IgG2a [12], FcgRIIb is a low-affinity inhibitory receptor binding to IgG2a and 2b, and FcgRIII binding to three IgG subclasses (IgG1, IgG2a, and IgG2b) and FcgRIV binding to IgG2a and IgG2b are low-affinity activating receptors. IgG2a are the most proinflammatory IgG molecules mainly binding to FcgRI in mice [13], and for this reason we did not investigate FcgRIV expression. The biological functions of FcgRs differ depending on their distinct affinity, cellular distributions, and biological characteristics. FcgRI and FcgRIII are reliant upon immunoreceptor tyrosine-based activating motifs (ITAM) and are recognized as activating receptors. In contrast, FcgRIIb is an inhibitory receptor with an immunoreceptor tyrosine-based inhibitory motif (ITIM)[31–33]. The deletion of activating receptors such as FcgRI and FcgRIII ameliorated immune responses resulting in autoimmunity and overt autoimmune disease, while the loss of the FcgRIIb inhibitory receptor exerted the opposite effect [11]. In contrast to a previous study by Lopez-Parra on diabetic Fcg receptor-deficient mice [26], our* in vivo* study demonstrated that FcgRIIb knockout diabetic mice exacerbated renal inflammation, fibrosis and the anomalous deposition of oxLDL, instead of having a renoprotective effect. The previous study reported FcgRIIb-knockout diabetic mice were prone to spontaneously developing diseases and susceptible to the induction of various autoimmune diseases [34]. Nevertheless, FcgRIII deficiency failed to protect mice from hyperglycemic and hypercholesterolemic renal injury. The upregulation of FcgRI in FcgRIIb knockout diabetic mice might counteract the effect of FcgRIIb deficiency on renal inflammation and fibrosis in hypercholesterolemic mice with diabetes. This was confirmed by our* in vitro* study showing that siRNA-FcgRI significantly downregulated the inflammatory and fibrosis of GMCs under high glucose and oxLDL-IC conditions. Unexpectedly, mice with FcgRIII deficiency (low-affinity activity receptor) did not show a lower susceptibility to diabetes compared with WT diabetic mice. FcgRI promotes disease in coordination with FcgRIII in diabetic mice. FcgRI binds immune complexes (ICs)[35] and augments renal injury because of the lack of FcgRIII [8].

Dyslipidemia is common in patients with DM, which leads to an increase in cholesterol and triglyceride levels. LDL oxidation is associated with the occurrence and development of diabetic nephropathy [36]. Our study revealed a higher deposition of oxLDL in the glomeruli of diabetic mice, with statistical differences between single FcgR deficient diabetic mice and WT diabetic mice, probably because single FcgR deficiency may alter the renal cell responses to immune complexes. Interestingly in our study, FcgRIIb or FcgRIII deficient HFD mice had higher levels of oxLDL protein and mRNA than WT HFD mice, whereas results for inflammatory and fibrosis, single FcgR deficient of HFD mice revealed no remarkable difference when compared with WT HFD mice. Lipid accumulation in nonadipose tissues is increasingly recognized to contribute to organ injury through a process termed lipotoxicity, it is just one element in diabetic nephropathy [37, 38]. Our results also showed that FcgRs activation by oxidized LDL-immune complexes is of pathologic importance in the acceleration of diabetic renal disease.

In our study, FcgRs, including FcgRI, FcgRIIb, and FcgRIII, were expressed in the kidneys of diabetic mice, and notably, the mRNA expressions of FcgRI and FcgRII were upregulated in FcgRIII-knockout diabetic mice. The mechanisms involved might include: (1) FcgRI is a high-affinity activity receptor that might play a major role in diabetic nephropathy by binding high numbers of IgG2 and oxidized LDL immune complexes that stimulate immune responses and downstream inflammation; (2) inhibitory receptor FcgRIIb was increased by feedback adjustment because the FcgRIII activation receptor was knocked out. Protein levels were not the same as mRNA levels, that has a more intrinsic and complex dependence, different regulation mechanisms acting on both the synthesized mRNA and the synthesized protein [39]; and (3) FcgRIIb-deficient mice did not express higher levels of FcgRIII compared with WT mice possibly because of a mutation in the gene promoter sequence[40, 41].. Whether differences in the expression of the FcgR gene are responsible for diabetic nephropathy remains to be determined.

In summary, different FcgRs have different roles in the development and progression of type 2 diabetes with diabetic nephropathy by regulating renal inflammation and fibrosis. The high-affinity activity receptor FcgRI has a leading role in aggravating renal lesions [8, 36, 42, 43]. These observations suggest that FcgRs are a novel target for therapeutic interventions to treat nephropathy in diabetic patients with dyslipidemia with high levels of circulating immune complexes.

## Figures and Tables

**Table 1:** General and biochemical parameters of diabetic, high-fat, and nondiabetic control mice.

Mice	WT			FcgRIIb-/-			FcgRIII-/-		
	Control	HFD	DM	WT	HFD	DM	Control	HFD	DM
Blood glucose (mmol/l)	9.7±0.54	14.8±0.4^a^	26.7±0.79^ab^	8.1±0.27	11.3±0.27^a^	27.4±0.92^ab^	7±0.31	17.7±0.45^a^	30.4±0.58^abc^
body weight (g)	24.6±0.38	32.9±0.78^a^	27.7±0.51^ab^	24.3±0.47	37.73±0.56^a^	30.5±0.78^a^	26.3±0.27	41±1.1^a^	33.5±0.51^abc^
Serum creatinine (umol/l)	10.67±0.44	15.97±0.53^a^	23.81±0.95^ab^	11.1±0.52	16.02±0.59^a^	26.7±0.39^abc^	13±0.43	17.28±0.61^a^	27.±0.71^abc^
BUN (mmol/l)	5.9±0.39	8.36±0.20^a^	9.74±0.12^ab^	6.60±0.36	7.60±0.19^a^	11.13±0.25^abc^	6.54±0.13	8.79±0.14^a^	11..51±0.18^abc^
LDL (mmol/l)	0.16±0.01	0.59±0.08^a^	0.52±0.04^a^	0.25±0.01	0.65±0.07^a^	0.71±0.02^ac^	0.25±0.02	0.72±0.06^a^	0.71±0.03^ac^
Chol (mmol/l)	2.01±0.08	4.41±0.08^a^	4.17±0.16^a^	2.16±0.09	5.58±0.18^a^	5.26±0.18^ac^	2.20±0.38	5.68±0.07^a^	5.15±0.13^ac^

Data are expressed as the mean ± SEM.

^a^p<0.05 versus respective nondiabetic control.

^b^p<0.05 versus respective HFD control.

^c^p<0.05 versus WT with DM.

## Data Availability

The data used to support the findings of this study are included within the article.

## Additional Points

Many previous studies have suggested that the immune system is involved in the development of diabetic nephropathy, and the Fc gamma receptors (FcgRs) are immune receptors that have an important role in the clearance of immune complexes. We believe that a key question will make it interesting to general readers, there is no evidence to clarify the individual role of FcgR family members in the pathogenesis of diabetic nephropathy, and our study is the first investigation of the involvement of FcgRI, FcgRIIb, and FcgR III in diabetic renal injury. In this study, we found that FcgRI knockdown downregulated inflammation and fibrosis. FcgRIIb knockout accelerated inflammation, fibrosis, and the anomalous deposition of oxLDL whereas FcgRIII deficiency failed to delay renal injury. Thus, these observations suggested that FcgRs might represent a novel target for the therapeutic intervention of diabetic nephropathy.

## Supplementary Materials

Supplementary 1 S1. Acridine orange fluorescent staining to mark the isolated glomeruli (scale ×400).

Supplementary 2 S2. Primer sequences.
