# Corrigendum to “FcgRIII Deficiency and FcgRIIb Defeciency Promote Renal Injury in Diabetic Mice”

**DOI:** 10.1155/2020/8385165

**Published:** 2020-07-01

**Authors:** Rui Zhang, Tingli Wang, Qinhua Yin, Junlin Zhang, Li Li, Ruikun Guo, Qianqian Han, Hanyu Li, Yiting Wang, Jiali Wang, Pramesh Gurung, Yanrong Lu, Jingqiu Cheng, Lin Bai, Jie Zhang, Fang Liu

**Affiliations:** ^1^Division of Nephrology, West China Hospital of Sichuan University, Chengdu, 610041 Sichuan, China; ^2^Key Laboratory of Transplant Engineering and Immunology, Ministry of Health, Regenerative Medicine Research Center, West China Hospital of Sichuan University, Chengdu, 610041 Sichuan, China

In the article titled “FcgRIII Deficiency and FcgRIIb Defeciency Promote Renal Injury in Diabetic Mice” [1], there was a spelling error in the title. The correct title is “FcgRIII Deficiency and FcgRIIb Deficiency Promote Renal Injury in Diabetic Mice”.
