# Supplementary figures and images for: FcgRIII Deficiency and FcgRIIb Defeciency Promote Renal Injury in Diabetic Mice
Source: Biomed Res Int. 2019 Aug 22;2019:3514574. doi: 10.1155/2019/3514574 (PMC6724446; doi:10.1155/2019/3514574)

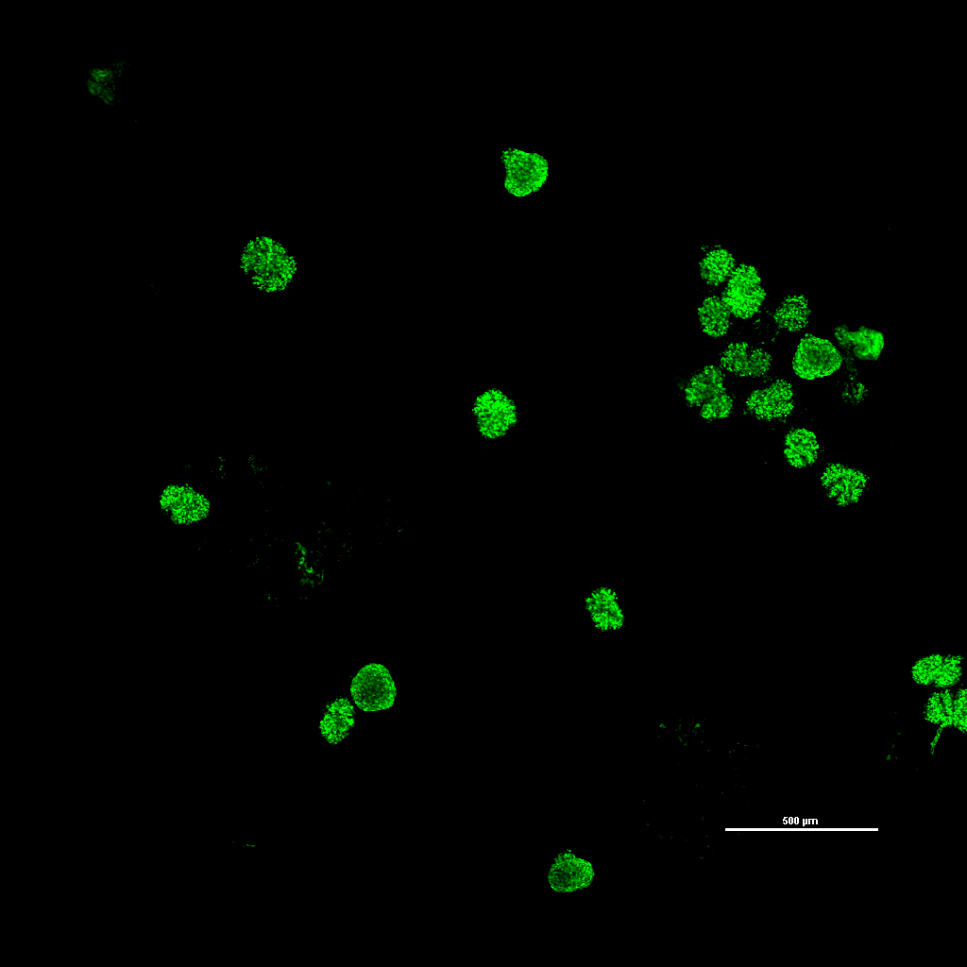


**S1.**

Supplement: Supplementary 1 — S1. Acridine orange fluorescent staining to mark the isolated glomeruli (scale ×400). [file 3514574.f1.docx]
