# Supplementary material for: FcgRIII Deficiency and FcgRIIb Defeciency Promote Renal Injury in Diabetic Mice
Source: Biomed Res Int. 2019 Aug 22;2019:3514574. doi: 10.1155/2019/3514574 (PMC6724446; doi:10.1155/2019/3514574)
Supplement: Supplementary 2 — S2. Primer sequences. [file 3514574.f2.docx]

| Gene | Sequences |
| --- | --- |
| TGF-β1 | Forward 5-AGTGGGCACTTGTCGTAC -3 |
|  | Reverse 5-AGCACAGCGTGGTTGA-3 |
| TNF-a | Forward 5-CACAGAAGTTCCCAAAT-3 |
|  | Reverse 5-CTGCCCTTCCTCCAT-3 |
| FcgRⅢ | Forward 5-CGCACTACCCGAAAC-3 |
|  | Reverse 5-AAGAAGAGCACGGAGAT-3 |
| FcgRⅡb | Forward 5-AAGACCCAGCAACTACAT-3 |
|  | Reverse 5-CAGGATTCCCATTCAC-3 |
| FcgRⅠ | Forward 5-GCCCAAAGCGTCCCTATT-3 |
|  | Reverse 5-ACTGTGGTCCATTATCCCG-3 |
| OxLDL | Forward 5-AAGACCCAGCAACTACAT-3 |
|  | Reverse 5-CAGGATTCCCATTCAC-3 |
| NFkb-p65 | Forward 5’-CCGACTTGTTTGGGTGATC- 3’ |
|  | Reverse 5’-AATCCGCGTGGAGGAAGAC-3’ |
| GAPDH | Forward 5’-CCTTCCGTGTTCCTAC-3’ |
|  | Reverse 5’-GACAACCTGGTCCTCA-3’ |

**S2**
